# Optimal Diagnosis Strategy via Functional Extremum Transformation and Fault Elimination Neural Network: A Minimum Diagnosis-Cost Approach for Complex Systems

**DOI:** 10.3390/s26092637

**Published:** 2026-04-24

**Authors:** Yuanzhang Su, Zhen Liu, Xiao Li, Xiaoting Tang, Min Wang, Jingyuan Wang

**Affiliations:** School of Automation Engineering, University of Electronic Science and Technology of China, Chengdu 611731, China; syz@uestc.edu.cn (Y.S.); 202222060306@std.uestc.edu.cn (X.L.); xiaotingtang@std.uestc.edu.cn (X.T.); mwang@uestc.edu.cn (M.W.); 202211060921@std.uestc.edu.cn (J.W.)

**Keywords:** diagnostic cost, minimum-cost diagnostic strategy generation problem (MDP), multipartite graph model, fault elimination neural network with intelligent algorithm (ENI), complex systems

## Abstract

For complex systems with complex structures and limited fault data, expert knowledge-based methods are often preferred. The multi-signal flow graph model is a representative qualitative approach, but generating a minimum-cost diagnostic strategy under this model becomes increasingly difficult as system scale grows, leading to excessive diagnostic cost or unacceptable computation time. To address this issue, this paper reformulates the minimum-cost diagnostic strategy generation problem (MDP) as a functional extremum value problem (FEVP) through a multipartite graph model, which decomposes the recursive strategy-generation process into deterministic combination and nondeterministic elimination. This reformulation is motivated by the observation that only a small fraction of generated fault subsets contributes to the optimal strategy. Based on this insight, a fault elimination neural network with an intelligent algorithm (ENI) structure is proposed to learn an elimination function that retains promising subsets while discarding redundant ones. The neural network scores candidate fault subsets from fixed-dimensional statistical features, while the intelligent algorithm optimizes the non-differentiable objective. Simulation and real-case results show that the proposed method reduces diagnostic cost by more than 30% while maintaining acceptable computation time, with greater advantages on larger-scale systems.

## 1. Introduction

### 1.1. Motivation

Fault diagnosis typically involves three main approaches: analytical model-based [1], signal processing-based [2], and expert knowledge-based methods [3]. For complex systems, the intricate structures make it difficult to develop accurate analytical models and acquire sufficient signal data. Consequently, expert knowledge-based diagnostic methods have emerged as the preferred choice [4,5]. The Multi-Signal Flow Graph model is a representative approach in this category [6,7]. It characterizes the relationships between faults and tests (“related” or “unrelated”) as a binary matrix, and isolates faults with a test strategy (diagnostic strategy) based on the matrix. This model closely reflects the real physical structure and exhibits convenience for construction and versatility in application, making it widely used in complex systems such as aerospace, telecommunications, and weaponry [8]. To enhance diagnostic efficiency, diagnosticians must solve a minimum-cost diagnostic strategy generation problem (MDP) under this model to minimize test costs [9]. However, with the growing complexity of system functionality and structure, the number of faults to be diagnosed has grown from under 20 to over 50, and the MDP has evolved from small-scale to large-scale problems. While existing methods prove adequate for small-scale MDPs, they lack scalability and perform poorly for large-scale problems. This has resulted in persistently high diagnostic costs for current complex systems, significantly impacting system operability and reliability [10].

### 1.2. Previous Research

According to the direction of strategy generation [11,12,13], the research can be categorized into top-down, horizontal, and bottom-up methods.

The top-down method starts from the full set of faults and searches downward until each fault is isolated. AO* is a typical class of direct backtracking algorithms that selects the length of Huffman coding, information gain, or information entropy as the heuristic function [14,15,16]. After the current node is expanded, the search path is corrected based on the synthesis cost. This ensures that each decision step is optimal, so the result is minimal. However, too many redundant computations leads to extremely long computation time. The rollout algorithm [17,18,19] sets parameters to adjust the frequency of backtracking based on AO*. It discards some operations to decrease the time complexity, but it will have a slight deviation from the ideal solution. Moreover, backtracking still takes a long time, and parameter setting becomes very difficult when facing complex systems. In the second class, indirect backtracking is performed via metaheuristic functions, where the diagnosis strategy is optimized via an intelligent algorithm. Ref. [20] encoded branches and optimized codes via a quantum genetic algorithm (QGA) to avoid the complex generation process. Ref. [21] utilized the self-adaptive binary particle swarm optimization (SADPSO) algorithm to find the optimal solution based on the greedy algorithm. Similarly, Refs. [22,23,24] used heuristic particle swarm optimization (HPSO), genetic algorithm, and cuckoo algorithm. These algorithms are limited by the randomness of intelligent algorithms and tend to fall into local optimal solutions. Unlike the above algorithms, the greedy algorithm [25] discards backtracking and selects the test with the least expansion cost for the current node; thus, it is extremely computationally efficient and has a similar cost to the ideal solution on small- and medium-scale systems. However, its performance is limited for large-scale systems because it lacks global consideration.

The horizontal method generates branches for each fault and integrates them into a strategy. Little research has been done on this topic. Ref. [26] generated each sequence independently according to the prior probability. Some costly errors are caused by ignoring the correlation among the branches, and the error further increases when the prior probabilities of each fault are close.

The bottom-up method arranges and combines all faults into multiple subsets and builds a full set. It enumerates all situations to obtain the least costly diagnosis strategy. Ref. [27] logically denies the possibility of partial permutations and proposes a look-ahead heuristic function to optimize the bottom-up process. Ref. [28] classified and selected the subsets according to the possibility of the Support Vector Machine (SVM) reducing unnecessary operations. The cost of these algorithms is almost optimal; however, when dealing with a complex system, the complexity of the enumeration operation is exponentially increased, and the computation time is too long.

Overall, existing methods can generate good diagnosis strategies for handling small-scale systems. However, when dealing with large-scale MDP of complex systems [29,30], some existing algorithms require unacceptable computation time due to combinatorial explosion, while others are prone to local optimal solutions due to the accumulation of errors from multiple decisions, resulting in a significant cost deviation between the generated strategy and the ideal strategy, leading to a large amount of additional test costs. To further improve the capability of fault-related analysis and optimization in complex systems, some recent studies have started to introduce deep-learning-based diagnosis, advanced signal-driven diagnosis, and neural-network-based fault estimation mechanisms [31,32]. These studies suggest that learning-based structures can provide additional flexibility and adaptability for complex fault-related problems.

### 1.3. Scope and Organization of the Paper

To overcome the above shortcomings of existing algorithms, this paper proposes a novel method for MDP based on fault combination and optimized elimination. Firstly, a multipartite graph model is established, which equates the generation of a strategy to a recursive process of faults from subsets to the entire set. The recursive function consists of a deterministic combination and a nondeterministic elimination. Therefore, the original optimization problem is transformed into a functional extremal value problem (FEVP) for elimination, which is much easier. Then, the elimination function is hard to analyze, so a fault elimination neural network with an intelligent algorithm (ENI) structure is proposed to decompose it into three parts: scoring function, retention, and criterion, and solution methods were provided separately. Finally, the optimal elimination function obtained is used to guide the recursive process. The experiment shows that the proposed method can generate a strategy with a lower test cost within an acceptable time. The innovations of this paper are as follows:

- The proposal of a multipartite graph model transforms the MDP into a FEVP, which is much easier to solve.
- As the optimization objective of FEVP is recursive, an ENI structure is established to overcome the difficulty in analyzing the unknown function.
- The neural network and intelligent algorithm in the ENI structure can be arbitrary.
- The proposed method can generate a diagnosis strategy with a lower test cost, and this advantage becomes prominent as the system size increases.

The paper is organized as follows. Section 2 describes the MDP based on the multi-signal flow graph model, and Section 3 elaborates on the proposed method. Simulations and experiments are presented in Section 4, and Section 5 provides the conclusion.

## 2. Problem Description

### 2.1. The Multi-Signal Flow Graph Model

The composition of the multi-signal flow graph model is as follows [33,34,35]:

1. A finite set of m system states, $S=\{s_{1},s_{2},\cdots ,s_{m}\}$, in which $s_{i}$ represent the different faulty states of the system;
2. The corresponding set of m known probabilities, $P=\{p_{1},p_{2},\cdots ,p_{m}\}$, satisfies $0\leq p_{i}\leq 1$, where $p_{i}$ is the a priori probability that the system is in state $s_{i}$, and $\sum_{i=1}^{m}p_{i}=1$;
3. A finite set of n binary tests to choose from, $T=\{t_{1},t_{2},\cdots ,t_{n}\}$;
4. The corresponding set of n test costs is $C=\{c_{1},c_{2},\cdots ,c_{n}\}$, where $c_{j}$ is the comprehensive cost of time and economy incurred when test $t_{j}$ is applied;
5. The dependency matrix $D=[d_{i,j}]$, where $d_{i,j}$ is 1 if test $t_{j}$ detects a failure state $s_{i}$ and 0 otherwise; to describe the distributions of 0 and 1 in D, r is defined as
  (1) $$r=\frac{1}{n}\sum_{j=1}^{n}\frac{\max\left(m-\sum_{i=1}^{m}d_{ij},\sum_{i=1}^{m}d_{ij}\right)}{\min\left(m-\sum_{i=1}^{m}d_{ij},\sum_{i=1}^{m}d_{ij}\right)}$$

**Figure 1:**
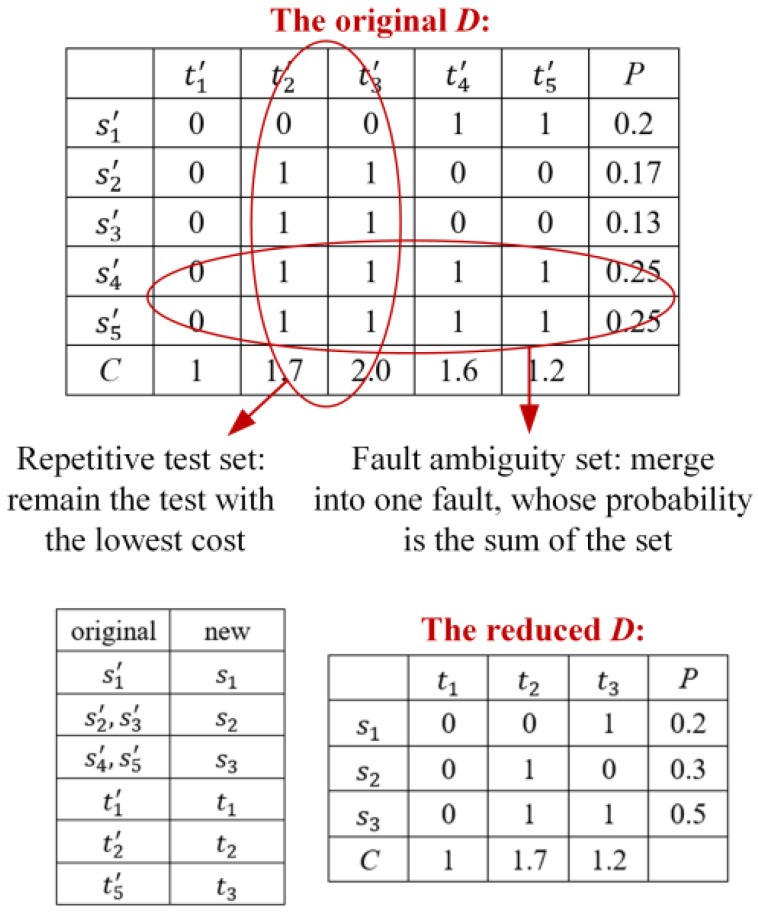
An example of dimensionality reduction.

In engineering applications, the functions of some tests are repetitive, and due to the limited types of tests that can be conducted, some fuzzy sets of faults that cannot be separated are formed. Therefore, it is necessary to reduce the dimensionality of the original D; an example is shown in Figure 1.

Notably, this paper discusses the reduced D and the diagnostic strategy with a 100% fault isolation rate, which satisfies

(2) $$\{\begin{array}{c} \forall s_{i},s_{k}\in S,\;\exists t_{j}\in T,\;d_{i,j}\neq d_{k,j}\\ \forall t_{j},t_{h}\in T,\;\exists s_{i}\in S,\;d_{i,j}\neq d_{i,h} \end{array}$$

D is obtained by sequential fault simulation. For example, $t_{1}$ is a voltage test performed at a certain point of the system, and the amplitude waveform of $t_{1}$ is ${wave}_{1}$ in the normal state, and the amplitude waveform of $t_{1}$ is ${wave}_{2}$ in the $s_{3}$ fault state in the simulation of $s_{3}$, if

(3) $$\frac{\left|{wave}_{1}-{wave}_{2}\right|}{{wave}_{1}}>emax$$

It indicates that if the occurrence of $s_{3}$ will affect $t_{1}$, then $s_{3}$ and $t_{1}$ are correlated, and $d_{31}=0$; otherwise, they are not correlated and $d_{31}=0$. emax is a specified upper limit of error, usually 5%.

Fault diagnosis is realized based on fusion analysis of the difference between normal and fault states and the correlation between tests and faults. If a test set $T^{\prime }=\{t_{j_{1}},t_{j_{2}},\ldots ,t_{j_{l}},\ldots ,t_{j_{L}}\}$ is selected by the system for testing, and its result is $\left\{z_{j_{1}},z_{j_{2}},\ldots ,z_{j_{l}},\ldots ,z_{j_{L}}\right\}$, $z_{j_{l}}=1$ indicates that the test data of $t_{j_{l}}$ is different from the normal data, and $z_{j_{l}}=0$ otherwise. Then the state of the system can be described as a fuzzy set S′, which satisfies

(4) $$S^{\prime }=\overset{L}{\underset{l=1}{⋃}}\left\{s_{i}|d_{ij_{l}}=z_{j_{l}}\right\}$$

**Figure 2:**
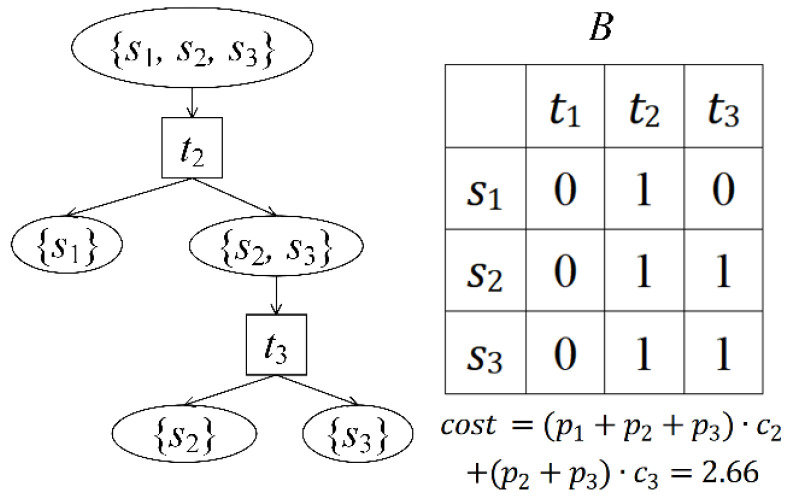
An example of a diagnosis strategy (B is defined in (5)).

For example, following the reduced D of Figure 1, if $T^{\prime }=\{t_{1},t_{2}\}$ and the test result is {0,1}, then $S^{\prime }=\{s_{2},s_{3}\}$, that is, the system either fails $s_{2}$ or $s_{3}$. The multi-signal flow graph model uses a diagnosis strategy (fault strategy) to describe the isolation of each fault and test order. As shown in Figure 2, the tester performs tests successively according to the instructions of the diagnosis strategy until the fault is located. $t_{2}$ is carried out first; if $z_{2}=0$, the fault is $s_{1}$, if $z_{2}=1$, the system status is $\left\{s_{2},s_{3}\right\}$ and $t_{3}$ is performed. If $z_{3}=0$, the fault is $s_{2}$; otherwise, it is $s_{3}$.

### 2.2. Combinatorial Explosion of MDP

One D can generate multiple diagnosis strategies, and expected cost is the key index to evaluate them:

(5) $$cost=\sum_{i=1}^{m}\sum_{j=1}^{n}c_{j}\cdot b_{ij}\cdot p_{i}$$

where $B=[b_{i,j}]$ is a binary matrix with dimensions of m×n; $b_{i,j}=1$ means that test $t_{j}$ is selected to identify fault state $s_{i}$; and otherwise, $b_{i,j}=0$. The lower the cost, the better the diagnosis strategy. Therefore, under the multi-signal flow graph model, the optimal fusion object problem can be described as an MDP:

(6) $$\{\begin{array}{c} \min cost\\ subject\;to\;\left(4\right) \end{array}$$

According to (2), a diagnosis strategy (binary strategy) must use m−1 tests; thus, the type of strategy ty is the cumulative product of the number of choices for each test. Taking S as the first layer, $s_{l}\left(u\right)$ represents the U-th fault subset of the L-th layer of the strategy; obviously, $u\leq 2^{l-1}$. l−1 tests are used, and n−(l−1) tests are unused from S to $s_{l}\left(u\right)$. The probability that $s_{l}\left(u\right)$ cannot be separated by any unused test is $r^{\left|s_{l}\left(u\right)\right|}+{\left(1-r\right)}^{\left|s_{l}\left(u\right)\right|}$, and this value is almost 0 when $|s_{l}\left(u\right)|>4$. If $m=2^{L}$ and the diagnosis strategy is a full binary strategy, it has L+1 layers; then, its type ${ty}_{0}$ can be approximated as

(7) $${ty}_{0}\approx {\left\{\left[1-r^{4}-{\left(1-r\right)}^{4}\right]\cdot \left(n-L+2\right)\right\}}^{\frac{m}{4}}\cdot {\left\{\left[1-r^{2}-{\left(1-r\right)}^{2}\right]\cdot \left(n-L+1\right)\right\}}^{\frac{m}{2}}\cdot \prod_{l=1}^{L-2}{\left(n-l+1\right)}^{2^{l-1}}$$

The combination of full binary strategies is simpler, so $ty>{ty}_{0}$. r=0.5 is taken to minimize (7) for estimation: when m=n=16, ${ty}_{0}\approx 2.6\times 10^{6}$; when m and n increase to 32 and 64, ${ty}_{0}$ increases to $9.3\times 10^{39}$ and $2.6\times 10^{101}$. As the complexity of the system continues to increase, the increase in m and n leads to an exponential increase in ty, making it extremely difficult to select the optimal one from so many types of diagnosis strategies. The deviation between the generated strategy of traditional algorithms and the ideal optimal solution will also increase, which is also called an NP-hard problem.

## 3. The Proposed Method

**Figure 3:**
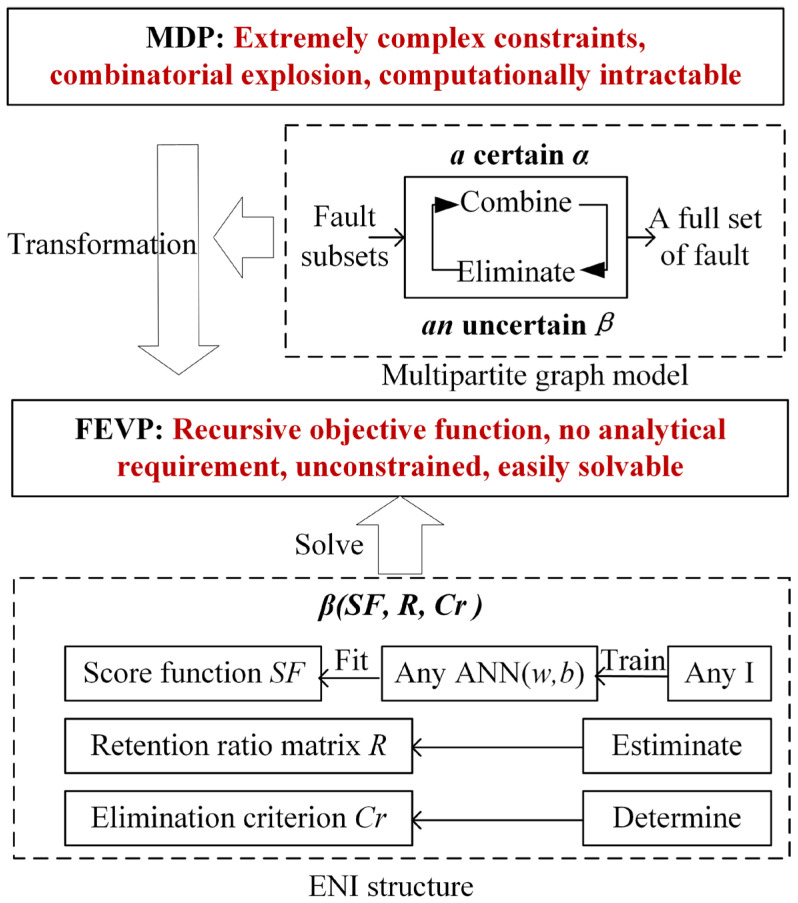
The concept diagram of the proposed method.

According to the analysis in the previous section, solving (6) is extremely hard due to the extremely complex constraints, and the increase in m and n leads to an NP-hard problem. As shown in Figure 3, first, a multipart graph model is established, and the generation of the diagnosis strategy is described as a recursive process of m−1 calls, from faults subsets to an entire set. The recursive function consists of a deterministic combination and a nondeterministic elimination. Based on this model, (6) is transformed into a FEVP for the elimination. As the elimination is only a part of the recursive function and has no constraints, the difficulty of solving this FEVP is much lower, and it is less affected by m and n.

This FEVP is relatively simple and different from typical common extremum problems [36]. The objective function is recursive and there is no analytical requirement for the unknown function. Therefore, an ENI structure is proposed to solve the optimal elimination function, which includes three parts: a score function, retention, and criterion. The score function is used to grade each element in the set to be eliminated based on their features. Retention refers to retaining elements with higher scores based on a ratio matrix R. The criterion Cr refers to the condition in which elimination is needed. R and Cr are estimated by the multipartite graph model. A neural network ANN(w,b) is used to fit the score function, and the optimal w,b are trained through an intelligent algorithm. Unlike in general optimization problems, the decision variables of the ENI are the parameters of the neural network and do not participate directly in the calculation of the fitness function. In addition, since ANN(w,b) is used only to fit the score function and an intelligent algorithm is used only to optimize w and b, they are not limited to a specific intelligent algorithm [37] or a neural network structure [38]. For different D, the generation methods of their diagnosis strategies are consistent, so a converged ANN(w,b) can be trained through a sufficient D, and the obtained (α,β) can be applied to another D.

### 3.1. The Multipartite Graph Model

A multipartite graph model is established and it includes $\left\{q_{i},p\left(q_{i}\right),cost\left(q_{i}\right),Q_{i}(u),ID\left(q_{i}\right),OD\left(q_{i},Q_{k}(u)\right)\right\}$ and two actions {α,β}.

$S_{i}$ represents a fault subset of S with a size i, satisfying $S_{i}\in 2^{s},\left|S_{i}\right|=i$. The reasonability criterion sequence ${tt}_{i}$ is a vector of $S_{i}$ with dimensions 1×n, whose j-th element satisfies

(8) $$\{\begin{array}{c} {tt}_{i,j}=1\;\;\forall s_{h}\in S_{i},\;d_{h,j}=1\\ {tt}_{i,j}=0\;\;\forall s_{h}\in S_{i},d_{h,j}=0\\ {tt}_{i,j}=x\;\;other \end{array}$$

where x refers to the irrelevant term. If $S_{i}$ satisfies (9), $S_{i}$ can be isolated from S through a finite number of tests; $S_{i}$ is reasonable and may become part of the optimal diagnosis strategy, denoted by $q_{i}$.

(9) $$\forall s_{h}\in S-S_{i},\exists t_{j},d_{h,j}+{tt}_{i,j}=1$$

According to (8), all $s_{i}$s are reasonable and satisfy ${tt}_{1,j}=d_{i,j}$. $p(q_{i})$ refers to the sum of prior probabilities of all faults in $q_{i}$, namely, $p\left(q_{i}\right)=\sum_{s_{h}\in q_{i}}p_{h}$. $cost(q_{i})$ represents the minimum cost required to isolate all elements in $q_{i}$, specifically, $cost\left(q_{1}\right)=0$ and $cost(q_{m})=J$. $Q_{i}$ refers to the set composed of all $q_{i}$. Specifically, $\left|Q_{1}\right|=m$, $Q_{m}=S$ and $|Q_{m}|=1$. $Q_{i}$ is eliminated to $Q_{i}(1)$ after $Q_{i}$ is generated, $Q_{i}(k-i)$ is eliminated to $Q_{i}(k-i+1)$ after $Q_{k}$ is generated, and $Q_{i}(m-i)$ is ultimately eliminated, where $Q_{i}(u)$ represents the result of $Q_{i}$ being eliminated for the U-th time. The family of sets $X_{k}$ is defined as $X_{k}=\{Q_{1}(k-1),Q_{2}(k-2),\ldots ,Q_{k-1}(1)\}$.

α represents a combination action, which refers to the process in which $X_{k}$ combines all elements in $Q_{1}(k-1)$ and $Q_{k-1}(1)$, $Q_{2}(k-2)$ and $Q_{k-2}(2)$, …, $Q_{\frac{k-1}{2}}(\frac{k+1}{2})$ and $Q_{\frac{k+1}{2}}(\frac{k-1}{2})$ (taking k as an odd number) and generate $Q_{k}$. If $q_{i}$ and $q_{k-i}(k>i)$ are disjoint fault subsets and $t_{j}$ meets

(10) $${tt}_{i,j}+{tt}_{k-i,j}=1$$

then $q_{k}=q_{i}\cup q_{k-i}$ exists, and $q_{i}$ and $q_{k-i}$ constitute a coupling of $q_{k}$. The cost of this coupling is

(11) $$cost\left(q_{i},q_{k-i}\right)=c_{j}+p\left(q_{i}\right)\cdot cost\left(q_{i}\right)+p\left(q_{k-i}\right)\cdot cost\left(q_{k-i}\right)$$

**Figure 4:**
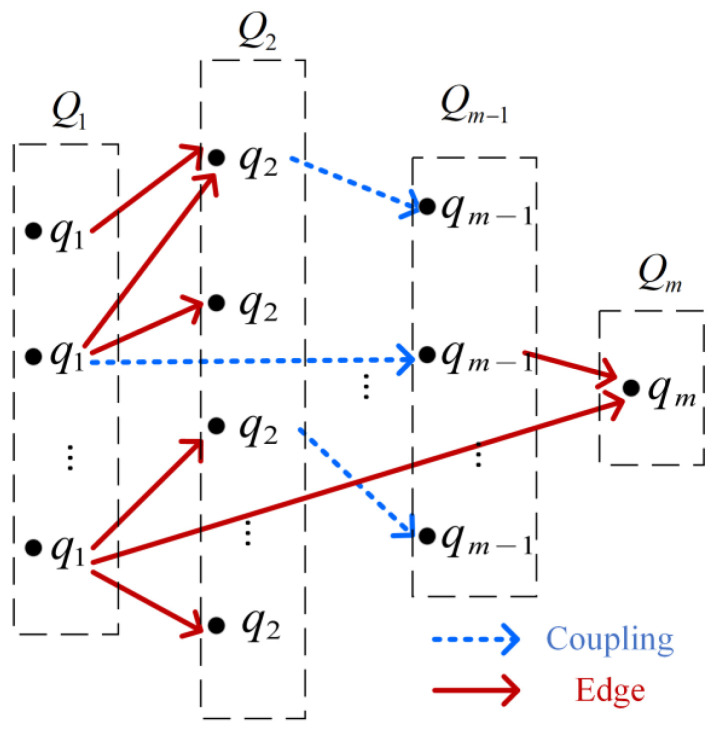
The m-partite Graph.

If the cost of this coupling is the least among all couplings, there is an edge between $q_{k}$ and $q_{i}$ and an edge between $q_{k}$ and $q_{k-i}$; then, $cost\left(q_{k}\right)=cost\left(q_{i},q_{k-i}\right)$. Considering $q_{i}$ as a vertex in a graph, $Q_{i}$ is a vertex set and $X_{k}$ constitute an k−1-partite graph [30]. As shown in Figure 4, edges and couplings exist only in different partite graphs. So α is a certain process selecting edges from all couplings according to (10) and (11); that is,

(12) $$Q_{k}=\alpha \left(X_{k}\right)=\left\{q_{k}|cost\left(q_{k}\right)=\min{⋃}_{i=1}^{\frac{k-1}{2}}{⋃}_{q_{i}\in Q_{i}\left(k-i\right),q_{k-i}\in Q_{k-i}\left(i\right)}cost\left(q_{i},q_{k-i}\right)\right\}$$

β represents an elimination action, which refers to the process that eliminates all sets of $X_{k}^{\prime }=\{X_{k},Q_{k}\}$ and obtains a new family of sets $X_{k+1}$. Due to the uncertainty of the elimination method, β is an uncertain process, that is,

(13) $$X_{k+1}=\beta \left(X_{k}^{\prime }\right)=\beta \left[X_{k},\alpha \left(X_{k}\right)\right]$$

$ID(q_{i})$ refers to the number of edges directing to $q_{i}$ and satisfies:

(14) $$\{\begin{array}{c} i=1,ID\left(q_{i}\right)=0\\ i\neq 1,ID\left(q_{i}\right)=2 \end{array}$$

**Figure 5:**
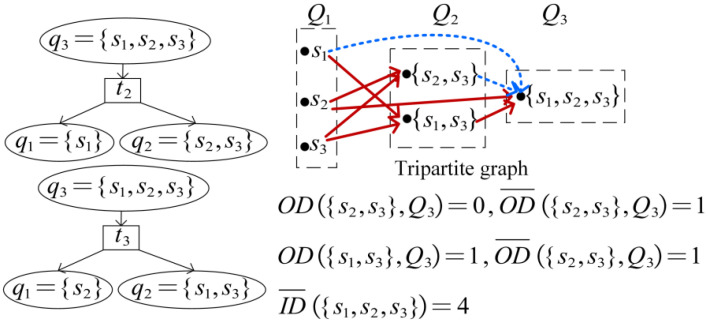
An example of a tripartite graph model.

$\bar{ID}\left(q_{i}\right)$ refers to the number of couplings directing to $q_{i}$ and indicates the number of combinations constituting $q_{i}.$ $\bar{ID}\left(Q_{i}\right)=\sum_{q_{i}\in Q_{i}}\bar{ID}\left(q_{i}\right)$ is the total number of couplings directing to $Q_{i}$. $OD[q_{i},Q_{k}\left(u\right)]$ and $\bar{OD}[q_{i},Q_{k}\left(u\right)]$ refer to the number of edges and couplings of $q_{i}$ directing to $Q_{k}\left(u\right)$. The reduced D in Figure 1 is taken as an example. As shown in Figure 5, $S=\{s_{1},s_{2},s_{3}\}$ can be composed of three combinations. Since the reasonability criterion sequence of $S_{2}=\{s_{1},s_{2}\}$ is 0×× there is no $t_{j}$ for $s_{3}$ to meet $d\left(3,j\right)+{tt}_{2,j}=1$, so $\left\{s_{1},s_{2}\right\}$ does not satisfy (10). S has only two couplings: $q_{1}=\left\{s_{1}\right\},q_{2}=\{s_{2},s_{3}\}$ and $q_{1}=\left\{s_{2}\right\},q_{2}=\{s_{1},s_{3}\}$. $cost\left(q_{i},q_{k-i}\right)$ are 2.66 and 2.39 according to (11), so there is an edge between S and $\left\{s_{2}\right\}$ and an edge between S and $\left\{s_{1},s_{3}\right\}$.

${best}_{i}$ is used to represent the set consisting of all $q_{i}$ that belong to the optimum diagnosis strategy. If $q_{k}$ can be expanded by a test, its subsets are denoted as $f(q_{k})$.

(15) $$f\left(q_{k}\right)=e_{1,k}\cdot q_{1}+e_{2,k}\cdot q_{2}+\cdots +e_{i,k}\cdot q_{i}+\cdots +e_{k-1,k}\cdot q_{k-1}$$

where $e_{i,k}$ refers to the expected number of $q_{i}$ generated by expanding $q_{k}$, specifically, $f\left(q_{1}\right)=2\cdot q_{1}$. $q_{k}$ is expanded into two subsets each time, so $\sum_{i=1}^{k-1}e_{i,k}=2$. Generally, $d_{i,j}$ is independent, so

(16) $$e_{i,k}=2\cdot \frac{C_{k}^{i}\cdot r^{i}{\cdot \left(1-r\right)}^{k-i}+C_{k}^{i}\cdot {\left(1-r\right)}^{i}\cdot r^{k-i}}{1-2\cdot {\left(1-r\right)}^{k}-2\cdot r^{k}}$$

Then, fault expansion can be represented in polynomial form by $f\left(q_{k}\right)$; if $q_{k}$ is expanded by l tests,

(17) $$F\left[f\left(q_{k}\right),l\right]=\underset{l\;times}{\underbrace{f\left(\ldots f\left(f\left(q_{k}\right)\right)\right)}}=\sum_{i=1}^{k-l}e_{i,k,l}\cdot q_{i}$$

where

(18) $$e_{i,k,l}=\sum_{\gamma }e_{k-a_{1},k}\cdot e_{k-a_{1}-a_{2},k-a_{1}}\cdot \cdots \cdot e_{i,i+a_{l}}$$

where γ refers to all the combinations that meet $k-i=a_{1}+a_{2}+\cdots +a_{l}\left(a_{1},a_{2},\ldots ,a_{l}\in N+\right)$ and $\left|\gamma \right|=C_{k-i-1}^{l}$. Completely isolating $q_{m}$ into $q_{1}$ requires at most m−1 iterations. For different i,k and l, each $e_{i,k,l}$ is independent,

(19) $$F\left[f\left(q_{m}\right),1\right]+F\left[f\left(q_{m}\right),2\right]+\cdots +F\left[f\left(q_{m}\right),m-1\right]=\sum_{i=1}^{m-1}\sum_{k=i+1}^{m}\sum_{l=1}^{m-1}e_{i,k,l}\cdot q_{i}$$

Therefore, the expected number of $q_{i}$ can be estimated as $\left|{best}_{i}\right|=\sum_{l=1}^{m-1}\sum_{k=i+1}^{m}e_{i,k,l}$. Taking an example of m=n=50,r=0.5, the estimation values of $\left|{best}_{i}\right|$ are shown in Table 1. According to the above analysis, it can be inferred that $\left|{best}_{i}\right|$ is extremely small, so the vast majority of $q_{i}$s do not participate in constructing the optimal diagnosis strategy. By eliminating these $q_{i}s$ properly and reasonably, many redundant operations can be reduced while minimizing the cost of the generated diagnosis strategy.

### 3.2. Transformation to an FEVP

**Figure 6:**
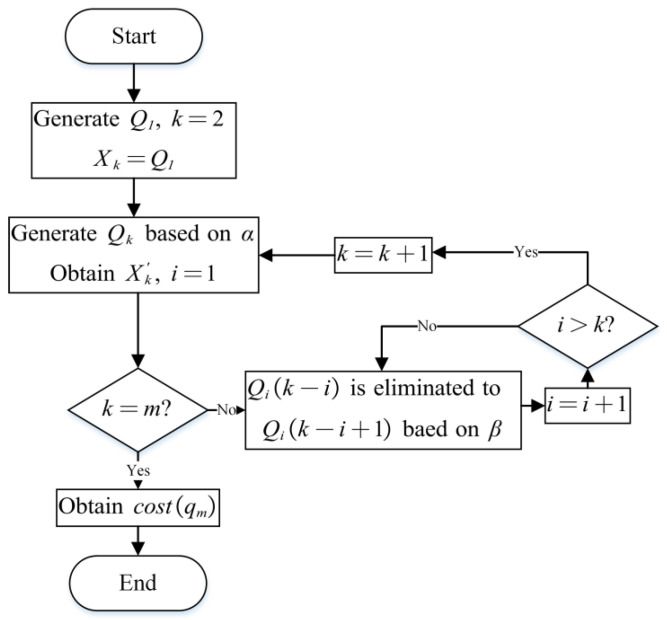
The state transition process.

As shown in Figure 6, based on (12) and (13), the generation of a diagnosis strategy is equated to a state transition process whose transition action includes combination and elimination. $X_{k}$ has two states, $\left\{{st}_{1},{st}_{2}\right\}$, where ${st}_{1}$ indicates that the elements belonging to the optimum diagnosis strategy of $X_{k}$ have not been mistakenly eliminated, namely, $\overset{k-1}{\underset{i=1}{⋃}}{best}_{i}\subseteq X_{k}$, and ${st}_{2}$ are the opposite state to ${st}_{1}$. $X_{k}^{\prime }$ also has two states: $\left\{{st}_{3},{st}_{4}\right\}$, where ${st}_{3}$ represents $\overset{k-1}{\underset{i=1}{⋃}}{best}_{i}\subseteq X_{k}^{\prime }$ and ${st}_{4}$ is the opposite state to ${st}_{3}$. TP is the state transition matrix of the four states, where ${tp}_{uv}$ refers to the probability of ${st}_{u}$ transferring to ${st}_{v}$. The state can only transfer between $X_{k}$ and $X_{k}^{\prime }$, so ${tp}_{12}={tp}_{21}={tp}_{34}={tp}_{43}={tp}_{11}={tp}_{22}={tp}_{33}={tp}_{44}=0$. Based on the deterministic combination, if the elements in ${best}_{i}$ are mistakenly eliminated, the father node $q_{k}(q_{k}\in {best}_{k})$ of $q_{i}$ cannot be generated. Therefore, ${tp}_{13}={tp}_{24}={tp}_{42}=1$ and ${tp}_{23}={tp}_{14}={tp}_{41}=0$ and ${tp}_{31}$ and ${tp}_{32}$ are two probabilities affected by β

(20) $$TP=\left[\begin{array}{cccc} 0 & 0 & 1 & 0\\ 0 & 0 & 0 & 1\\ {tp}_{31}\left(\beta \right) & {tp}_{32}\left(\beta \right) & 0 & 0\\ 0 & 1 & 0 & 0 \end{array}\right]$$

According to (20), the optimal solution cannot be obtained once the state transfers to ${st}_{2}$ or ${st}_{4}$. The ideal β meets ${tp}_{31}\left(\beta \right)=1-{tp}_{32}\left(\beta \right)=1$ and can eliminate all the redundant $q_{i}$s and retain all the elements of ${best}_{i}$, making the calculation speed extremely fast and $cost(q_{m})$ optimal. Therefore, the generation of a diagnosis strategy can be described as a recursive process of m−1 calls, and the original optimization problem (6) is transformed into an FEVP:

(21) $$\{\begin{array}{c} \min cost\left(Q_{m}\right)\\ Q_{k}=\alpha \left[\beta \left(X_{k},Q_{k-1}\right)\right] \end{array}$$

The objective function of (21) is recursive and the unknown function is a nondeterministic elimination. Because the elimination is only a part of the objective function and has no constraints, (21) is easier to solve.

### 3.3. ENI Structure

**Figure 7:**
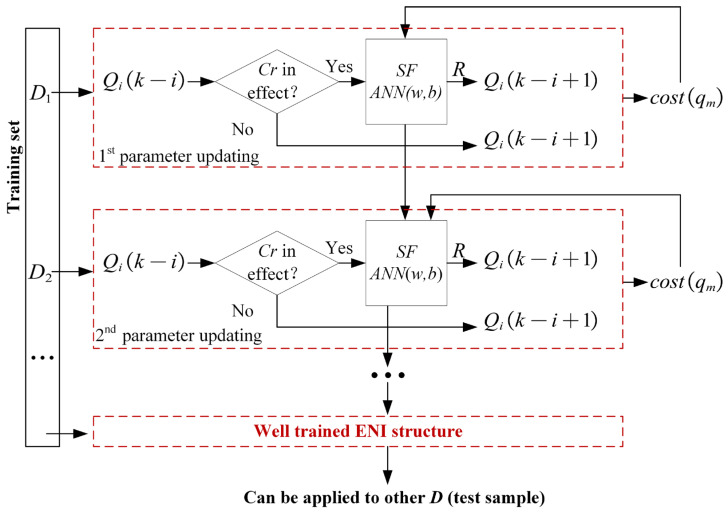
A schematic diagram of the ENI structure.

An ENI structure is proposed to determine the optimal β, as shown in Figure 7, β is described by SF,R and Cr. When the elimination criterion Cr is in effect, all $q_{i}$s of $Q_{i}\left(k-i\right)$ are scored according to SF and those with higher scores are retained, with a retention ratio $R_{i,k}$, that is:

(22)    Qi(k−i+1)=Qi(k−i)    Cr is not in effect  {|Qi(k−i+1)|=Ri,k⋅|Qi(k−i)|∀qi∈Qi(k−i)−Qi(k−i+1)∀qh∈Qi(k−i+1)SF(qi)<SF(qh)Cr is in effect

A neural network is used to fit the score function of $q_{i}$. The input features are the knowledge of $q_{i},Q_{k},D$ and C, including $p\left(q_{i}\right),cost\left(q_{i}\right),ID\left(q_{i}\right),\bar{ID}\left(q_{i}\right),OD\left(q_{i},Q_{k}\right),\bar{OD}\left(q_{i},Q_{k}\right),r,C$ and the prediction cost of $S-q_{i}$ (obtained through the greedy algorithm based on an entropy heuristic function [18]). The fitness value $cost(q_{m})$ of the current score function can be obtained based on the multipartite graph model. Initially, w and b are random, so the score function is unreasonable and leads to a high $cost(q_{m})$. Then, w and b are optimized continuously by the update method based on an intelligent algorithm. Finally, a score function that can generate a lower $cost(q_{m})$ is trained. For different D, the generation methods of their fault subsets according to (12) and the calculations of their fitness values according to (11) are consistent, so ANN(w,b) trained on one D can be transferred to another D to continue training until ANN(w,b) converges. A sufficient number of D matrices are used for training, and the well-trained ANN(w,b), i.e., the optimized β(SF,R,Cr), can be applied to other D.

It should be noted that (w,b) are not trained by standard gradient descent, because the fitness function of ENI is not differentiable with respect to ANN parameters. After ANN(w,b) outputs the scores of all $q_{i}s$, the following ranking, retention, elimination, and multipartite-graph reconstruction are all discrete operations. As a result, the mapping from (w,b) to $cost(q_{m})$ is nonanalytic and nondifferentiable, so backpropagation cannot be directly applied. Therefore, ANN(w,b) is used to fit the scoring function, while intelligent algorithms are adopted for parameter optimization under the final diagnosis-cost objective.

To avoid ambiguity, the input of the ANN is not the raw dependency matrix D itself. Instead, for each candidate fault subset to be evaluated during elimination, a fixed-length feature vector is constructed from aggregated statistical features derived from the current subset and its diagnostic context. Therefore, the input dimension of the ANN remains constant and is fully decoupled from the system scale (m,n). In other words, the ANN does not learn a direct mapping from a variable-sized matrix to an elimination decision; rather, it learns a mapping from a scale-invariant statistical representation of a candidate subset to its elimination score. This provides the mathematical basis for the transferability of the ENI structure across different problem sizes.

### 3.4. Training and Convergence of ANN(w,b)

For any k and i, the statistics and operation methods of the features are consistent when $Q_{i}\left(k-i\right)$ is eliminated; therefore, $Q_{i}\left(k-i\right)$ uses the same neural network ANN(w,b). The ANN(w,b) training process is stated in Algorithm 1.
**Algorithm 1** The ANN(w,b) training process1Select any intelligent algorithm and neural network structure.2Set the number of particles and the number of iterations as $z_{max}$ and ${it}_{max}$. 3Initialize $W=\{w_{1},w_{2},\ldots ,w_{z_{max}}\}$ and $b=\left\{b_{1},b_{2},\ldots ,b_{z_{max}}\right\}$, where $w_{z}$ and $b_{z}$ represent the weight of the neural network for the z-th particle. Randomly generate one D.4for $it=1:{it}_{max}$ do5
Update W and b using intelligent algorithms.6
for $z=1:z_{max}$ do7

Use $ANN(w_{z},b_{z})$ to calculate $cost(q_{m})$ of D based on (11) and (12).8
end9
Update $w_{z}$ and $b_{z}$ using the intelligent algorithms based on $cost(q_{m})$ of all particles. Record the smallest $cost(q_{m})$ in this iteration as $cost\left(q_{m},it\right)$.10end

If $cost\left(q_{m},it\right)=cost\left(q_{m},1\right)$, this indicates that training on this D did not improve W or b. If this situation occurs continuously on ten D, then W and b have converged, and

(23) $$w,b=arg\;\underset{W,b}{\min}\;cost\left(q_{m}\right)$$

### 3.5. Estimation of R

To quantify the proportion of elimination, the retention ratio R (an upper triangular matrix) is defined, where $R_{i,k}$ represents that the proportion of elimination is $1-R_{i,k}$ when eliminating $Q_{i}\left(k-i\right)$. Ideally, the final retention of $Q_{i}$ is ${best}_{i}$. Considering the errors caused by the estimation and fitting function, the actual retention is θ times that of $\left|{best}_{i}\right|$; that is,

(24) $$R_{i,i}\cdot R_{i,i+1}\cdot \cdots \cdot R_{i,m-1}\cdot \left|Q_{i}\right|=\theta \cdot \left|{best}_{i}\right|$$

The value of θ is discussed in Experiment 1. If no elimination is implemented, the probability of taking each value for ${tt}_{i,j}$ of any $S_{i}$ is:

(25) $$\{\begin{array}{c} p\left({tt}_{i,j}=0\right)=p\left(\forall s_{h}\in S_{i},d_{h,j}=0\right)=r^{i}\\ p\left({tt}_{i,j}=1\right)=p\left(\forall s_{h}\in S_{i},d_{h,j}=1\right)={\left(1-r\right)}^{i}\\ p\left({tt}_{i,j}=x\right)=1-r^{i}-{\left(1-r\right)}^{i} \end{array}$$

According to (8) and (9), if $s_{h}\in S-S_{i}$ exists and all the non x terms of ${tt}_{1}$ are the same as ${tt}_{i}$, then ${tt}_{i}$ cannot meet the reasonability criterion. Therefore, the scale of $Q_{i}$ without elimination (${\left|Q_{i}\right|}^{\prime }$) can be estimated as follows:

(26) $${\left|Q_{i}\right|}^{\prime }\approx C_{m}^{i}\cdot {\left[1-\prod_{j=1}^{n}p\left({tt}_{1,j}={tt}_{i,j}\neq x\right)\right]}^{m-i}=C_{m}^{i}\cdot {\left\{{1-\left(r^{i+1}+{\left(1-r\right)}^{i+1}\right)}^{n}\right\}}^{m-i}$$

According to (12), the number of couplings $Q_{k}$ generated by $Q_{i}$ or $Q_{k-i}$ is estimated as follows:

(27) $$\sum_{q_{i}\in Q_{i}}\bar{OD}\left(q_{i},Q_{k}\right)={\left|Q_{i}\right|}^{\prime }\cdot {\left|Q_{k-i}\right|}^{\prime }\cdot \frac{C_{m-i}^{k-i}}{C_{m}^{k-i}}\cdot \frac{\left|Q_{k}\right|\prime }{C_{m}^{k}}$$

where $C_{m-i}^{k-i}/C_{m}^{k-i}$ refers to the probability that $q_{i}$ and $q_{k-i}$ are disjointed and ${\left|Q_{k}\right|}^{\prime }/C_{m}^{k}$ refers to the probability that $S_{i}=q_{i}$. If the elimination is implemented, $S_{k}$ is separated into $S_{i}$ and $S_{k-i}$, and the probability that both are reasonable is $u_{i,k-i}$.

(28) $$u_{i,k-i}\approx \frac{\left|Q_{i}\left(k-i\right)\right|}{C_{m}^{i}}\cdot \frac{\left|Q_{k-i}\left(i\right)\right|}{C_{m}^{k-i}}$$

$S_{i}$ and $S_{k-i}$, respectively, have $C_{m}^{i}$ and $C_{m}^{k-i}$ combinations. If all the separating forms of $S_{k}$ are unreasonable, then $S_{k}$ is unreasonable. Thus, $\left|Q_{k}\right|$ can be estimated as the product of combination type $C_{m}^{k}$ and the probability of reasonability.

(29) $$\left|Q_{k}\right|\approx C_{m}^{k}\cdot \left[1-\prod_{i=1}^{\frac{k-1}{2}}{\left(1-u_{i,k-i}\right)}^{C_{k}^{i}}\right]$$

Corresponding to (27), the number of couplings $Q_{k}$ generated by $Q_{i}(k-i)$ or $Q_{k-i}(k)$ is estimated as follows:

(30) $$\sum_{q_{i}\in Q_{i}\left(k-i\right)}\bar{OD}\left(q_{i},Q_{k}\right)=\left|Q_{i}\left(k-i\right)\right|\cdot \left|Q_{k-i}\left(i\right)\right|\cdot \frac{C_{m-i}^{k-i}}{C_{m}^{k-i}}\cdot \frac{\left|Q_{k}\right|}{C_{m}^{k}}$$

To minimize the change in combination type caused by elimination, the proportion of the combination form of $q_{i}$ and $q_{k-i}$ must remain unchanged; that is, for ∀i<k,

(31) $$\frac{\sum_{q_{i}\in Q_{i}\left(k-i\right)}\bar{OD}\left(q_{i},Q_{k}\right)}{\sum_{q_{i}\in Q_{i}}\bar{OD}\left(q_{i},Q_{k}\right)}=b$$

where b is a constant. According to (24), (27) and (30),

(32) $$\frac{\left|Q_{i}\right|\cdot \left|Q_{k-i}\right|\cdot \left|Q_{k}\right|}{\left|Q_{i}\right|\prime \cdot \left|Q_{k-i}\right|\prime \cdot \left|Q_{k}\right|\prime }\cdot \prod_{h=i}^{k-1}R_{i,h}\cdot \prod_{h=k-i}^{k-1}R_{k-i,h}=b$$

Therefore, $R_{i,k}$ is any feasible solution that satisfies (24) and (32).

### 3.6. Determination of Cr

Eliminating $X_{k}^{\prime }$ can reduce the time required for diagnosis strategy generation based on the multipartite graph model. However, the steps required for β(SF,R,Cr), including calculating the statistics of the features, scoring each $q_{i}$ by ANN(w,b) and sorting, increase the operation time. It is necessary to analyze whether eliminating $X_{i}^{\prime }$ would be beneficial for improving operation efficiency after $Q_{i}$ is generated. According to (29), $\bar{ID}\left(Q_{k}\right)=\sum_{i=1}^{\frac{k-1\;}{2}}\sum_{q_{i}\in Q_{i}\left(k-i\right)}\bar{OD}\left(q_{i},Q_{k}\right)$, so the time needed to generate $\left\{Q_{i+1},Q_{i+2},\ldots ,Q_{m}\right\}$ is:

(33) $$time={∆}_{1}\cdot \sum_{k=i+1}^{m}\sum_{i=1}^{\frac{k-1\;}{2}}\sum_{q_{i}\in Q_{i}\left(k-i\right)}\bar{OD}\left(q_{i},Q_{k}\right)$$

where ${∆}_{1}$ refers to the time required to generate a coupling. If elimination is not executed, $Q_{i}\left(k-i+1\right)=Q_{i}\left(k-i\right)$. If elimination is executed, $\left|Q_{i}\left(k-i+1\right)|=R_{i,k}\cdot |Q_{i}\left(k-i\right)\right|$. ${time1}_{i}$ is the difference between the two results calculated based on (32) and refers to the temporal benefit of elimination. ${time2}_{i}$ is used to indicate the time needed to eliminate $X_{k}^{\prime }$, which is proportional to the number of all fault subsets contained in $X_{k}^{\prime }$:

(34) $${time2}_{i}={∆}_{2}\cdot \sum_{h=1}^{i}\left|Q_{h}\left(i-h\right)\right|$$

where ${∆}_{2}$ refers to the time required for $q_{i}$ to be scored by a neural network and sorted. ${∆}_{1}/{∆}_{2}$ is related to m,n and obtained through Monte Carlo simulation, as shown in Table 2.

Therefore, the criterion for eliminating the virus was ${time1}_{i}>{time2}_{i}$. If the criterion loses efficacy after $Q_{i}$ is generated, then ${time1}_{i}\leq {time2}_{i}$. After $Q_{k}$ is generated, the number of sets to be generated is reduced, so ${time1}_{k}<{time1}_{i}$. Moreover, as the number of generated sets increases, ${time2}_{k}>{time2}_{i}$. Thus, ${time1}_{k}<{time2}_{k}$, and each fault set will no longer be eliminated after the criterion loses efficacy.

### 3.7. Computational Complexity Analysis

To provide a more rigorous quantitative comparison, the computational complexity of the proposed method is analyzed from the two actions in the multipartite graph model, namely, combination α and elimination β. Let

(35) $$N_{k}=\sum_{\left\{i=1\right\}}^{k-1}\left|Q_{i\left(k-i\right)}\right|+\left|Q_{k}\right|$$

denote the total number of candidate subsets involved when generating the (k)-th layer. In the combination stage, all candidate couplings between $Q_{i}\left(k-i\right)$ and $Q_{k-i}\left(i\right)$ must be checked according to (10), and their costs must be evaluated according to (11). Therefore, the complexity of α at layer k is

(36) $$T_{\alpha }\left(k\right)=O\left(n\sum_{i=1}^{\left\lfloor \left(k-1\right)/2\right\rfloor }\left|Q_{i}\left(k-i\right)\right|\left|Q_{k-i}\left(i\right)\right|\right)$$

After $Q_{k}$ is generated, the elimination action β computes the features of all subsets in $X_{k}^{\prime }=X_{k},Q_{k}$, scores them by ANN, and sorts them for retention. If the forward complexity of ANN is denoted by $C_{ANN}$, then the complexity of β at layer k is

(37) $$T_{\beta }\left(k\right)=O\left(N_{k}C_{ANN}+N_{k}\log N_{k}\right)$$

Accordingly, the total complexity of one strategy-generation process is

(38) $$T_{\mathrm{prop}}=O\left(\sum_{k=2}^{m}\left[n\sum_{i=1}^{\left\lfloor \left(k-1\right)/2\right\rfloor }\left|Q_{i}\left(k-i\right)\right|\left|Q_{k-i}\left(i\right)\right|+N_{k}C_{ANN}+N_{k}\log N_{k}\right]\right)$$

If no elimination is performed, i.e., all subsets are retained, then $Q_{i}\left(u\right)=Q_{i}$, and the dominant term degenerates to

(39) $$T_{\mathrm{no}-\beta }=O\left(\sum_{k=2}^{m}n\sum_{i=1}^{\left\lfloor \left(k-1\right)/2\right\rfloor }\left|Q_{i}\right|\left|Q_{k-i}\right|\right)$$

Therefore, the main contribution of ENI is to reduce $\left|Q_{i}\right|$ to $\left|Q_{i}u\right|$, so that the dominant coupling-enumeration term is significantly compressed in subsequent layers. Although β introduces additional scoring and sorting overhead, this extra cost is much smaller than the reduction in redundant couplings for large-scale systems.

## 4. Experiments

To verify the effectiveness of the proposed method, we use a sufficient D of different sizes as the training set until ANN(w,b) converges, and the trained β are applied to three experiments. Experiment 1 simulates the impact of θ. Experiment 2 compared the results of the proposed method with those of existing algorithms at different scales. The performances of different combinations of neural network structures and intelligent algorithms on the ENI are also discussed. Experiment 3 selected two typical complex circuit systems as real cases (a control moment gyro system [20] and a two-channel multiple-input multiple-output (MIMO) terminal system).

Four comparative algorithms are selected, and their parameters are adjusted to the best values. The population size and number of iterations of the QGA [20] and HPSO [22] are 200 and 200, respectively, and both of their mutation rates are less than 5%. The third and fourth algorithms selected are the Growing [26] and Hybrid [28] algorithms, respectively. The D used in Experiments 1 and 2 were randomly generated according to r=0.5, with C and P following a uniform distribution from 0 to 2 and a normalized uniform distribution from 0 to 1. The software used for the experiments was MATLAB2022b in Windows 11, and the processor used was an AMD R9 CPU @ 2.50 GHz.

### 4.1. Sensitivity Analysis

The cost of the generated strategy is related to θ and $r_{g}$ when applying the trained β to test samples.

When θ is small, there may be errors in the estimation of $\left|{best}_{i}\right|$, resulting in unreasonable elimination of $Q_{i}$, and the reserved fault set cannot be used to generate $q_{m}$. When θ gradually increases to a certain value to meet $X_{m}^{\prime }\cap \{\overset{m}{\underset{i=1}{⋃}}{best}_{i}\}\approx \{\overset{m}{\underset{i=1}{⋃}}{best}_{i}\}$, a better test cost can be generated (denoted as cost convergence). If θ continues to increase, the additional $q_{i}$ retained does not participate in constructing the optimal diagnosis strategy, meaning that $X_{m}^{\prime }\cap \{\overset{m}{\underset{i=1}{⋃}}{best}_{i}\}$ remains almost unchanged and that the cost remains largely unchanged.

In addition to θ, another practical parameter is introduced in the implementation of the baseline GA strategy generator, namely the proportional factor $r_{g}$, which controls the optimization budget by setting the iteration number and population size as $IT=POP=m\cdot r_{g}$. Since the system scale increases with m, using $r_{g}$ instead of directly assigning fixed values to IT and POP provides a normalized way to compare different scales under comparable computational intensity.

**Figure 8:**
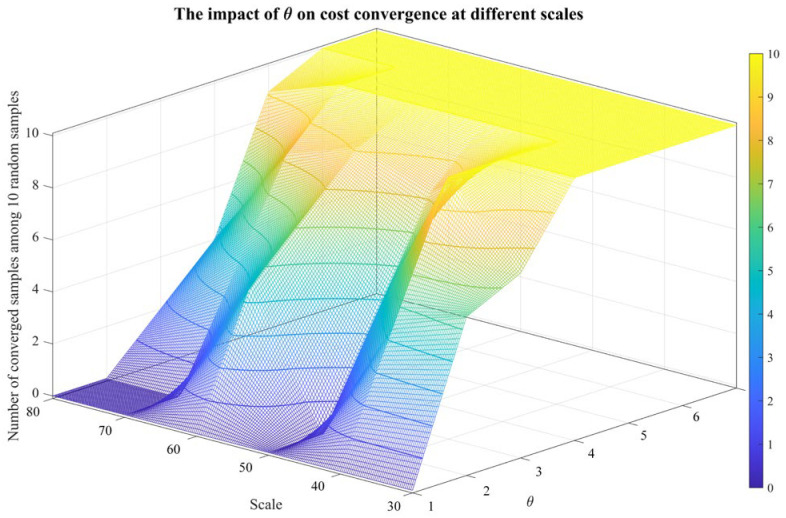
The impact of θ on cost convergence at different scales.

**Figure 9:**
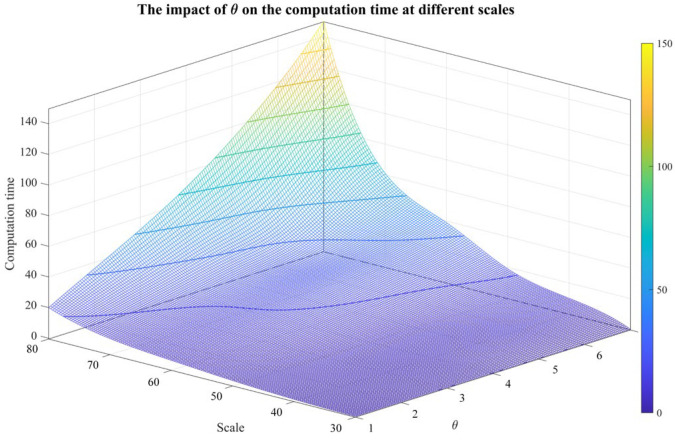
The impact of θ on the computation time at different scales.

For sensitivity analysis of θ, 10 samples for each scale range from 30×30 to 80×80 are randomly generated. Then, the number of converged samples among 10 random samples is counted, and the average computation time is recorded for different θs. The results are shown in Figure 8 and Figure 9.

**Figure 10:**
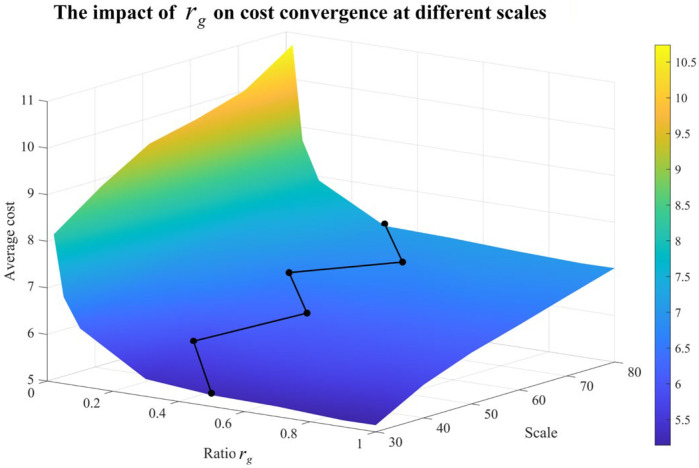
The impact of $r_{g}$ on cost convergence at different scales (Convergence points of different scales are connected by the black line).

**Figure 11:**
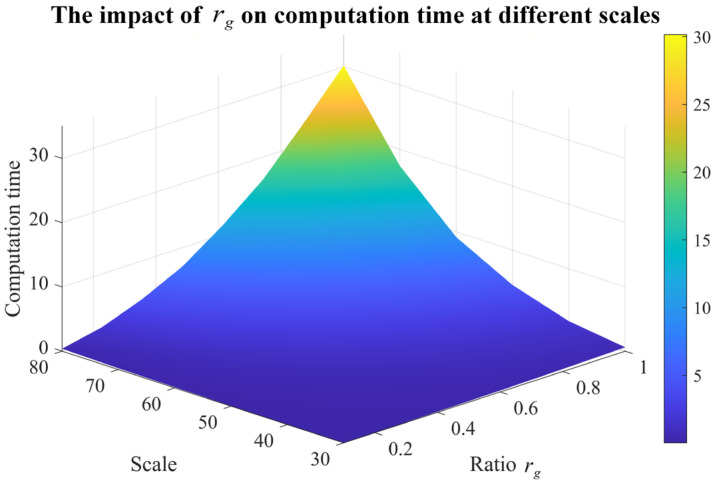
The impact of $r_{g}$ on the computation time at different scales.

For sensitivity analysis of $r_{g}$, 10 samples for each scale range from 30×30 to 80×80 are randomly generated. The average cost was calculated over 10 randomly generated samples. Then, for each fixed system scale, the value of $r_{g}$ at which the cost tended to converge as $r_{g}$ varied was identified, and the corresponding convergence point was marked in black. And the average computation time is recorded for different $r_{g}$s. The results are shown in Figure 10 and Figure 11.

The larger the system scale is, the larger its corresponding $\left|Q_{i}\right|$. Due to the randomness of intelligent algorithms, there is a small deviation between the trained β and the ideal β, which increases the difficulty of retaining $Q_{i}(m-1+i)\cap {best}_{i}$. Therefore, a greater θ is required to converge, and the computation time also increases.

When $r_{g}$ is too small, the search space cannot be explored sufficiently, and the generated diagnosis strategy is likely to have a relatively high cost. As $r_{g}$ increases, the search becomes more adequate, and the diagnosis cost decreases accordingly. However, when $r_{g}$ exceeds a certain range, the improvement in cost becomes marginal. In the experiments, it was observed that when $r_{g}$ is around 0.5, the cost tends to become nearly stable for most scales.

### 4.2. Performance Differences of Algorithms at Different Scales

Ten samples at each scale are generated and the average computation time and expected cost of the proposed method with those of existing algorithms are compared. To analyze the impact of the neural network and intelligence algorithm on the ENI, three neural network structures are selected: a multilayer perceptron (MLP) with one or two hidden layers and a long short-term memory (LSTM) network. Three intelligent algorithms are selected: particle swarm optimization (PSO), the genetic algorithm (GA), and the cuckoo bird algorithm (CS). $\theta ,r_{g}$ is set based on the results of Experiment 1 while considering both computation time and convergence. The experimental results for the 9 ENIs and comparative algorithms are shown in Table 3 and Table 4, where the cost of PSO_MLP1 is given in units of “1”.

Due to the long computation time, Hybrid is almost impossible to carry out on a scale greater than 70×70. In terms of cost, the proposed method and Hybrid method yield lower costs at all scales, and our method outperforms the other methods. At 80 × 80, the test cost is only 69.4% of the Growing algorithm. In terms of time, the Growing algorithm has the shortest time, and the proposed method is shorter than the QGA, HPSO, and Hybrid algorithms at small to medium scales. As the scale increases, the computation time of the proposed method can reach 97 s at 80×80, which is shorter than that of the Hybrid. However, since the diagnosis strategy is generated during the design phase and does not have real-time requirements, 97 s is acceptable. In addition, any neural network structure has the ability to fit the score function theoretically, and its parameters are optimized by intelligent algorithms; thus, the optimization ability of these ENIs is the same. The complex structure will expand the optimized decision space, so MLP2 and LSTM will slightly increase the computational time, but the costs are almost the same.

To clarify the robustness of the proposed ENI structure, three aspects should be emphasized. First, ENI does not operate directly on the raw dependency matrix D. Instead, it learns a scoring function from fixed-dimensional aggregated features extracted from the current candidate fault subset. Therefore, the input dimension of the network is decoupled from the system scale (m,n), and the statistical meaning of the input remains consistent across different problem sizes. This scale-decoupled representation improves the stability when it is transferred across different dependency matrices. Second, the role of ENI is not to generate a complete diagnosis strategy directly, but to rank candidate fault subsets. As a result, ENI mainly needs to preserve a reliable relative ordering among candidate subsets, rather than predict an exact physical quantity, which reduces the sensitivity of the final strategy cost to fluctuations in network architecture and initialization. Third, the neural network and the intelligent algorithm play different roles in ENI: the former approximates the scoring function, whereas the latter searches for favorable parameters under a non-differentiable objective. This separation makes the ENI framework compatible with different neural-network structures and different intelligent algorithms.

The hyperparameter selection is also not arbitrary, but consistent with the functional decomposition of ENI. For the neural network, the input is a low-dimensional statistical feature vector, and the task is to learn a coarse-grained priority score for candidate subsets. Therefore, a shallow MLP is already expressive enough for this purpose. More complex architectures enlarge the parameter space, but do not necessarily improve the final diagnosis cost, while they do increase the training and inference overhead. For the intelligent algorithm, the population size and the number of iterations should balance search sufficiency and offline computational burden: too small a setting leads to insufficient exploration, whereas too large a setting yields marginal performance gain at the cost of substantially increased computation time.

### 4.3. Real-Case Verification

The first case is a control moment gyro system [20], which is an important execution structure for controlling spacecraft flight attributes. This system consists of 28 different tests and 36 failure states (reduced D), and all the test costs are 1. Under the premise of ensuring a 100% fault isolation rate, a diagnostic strategy with minimal cost needs to be designed for the system. Based on Experiments 1 and 2, PSO_MLP1 is selected with θ=4 and $r_{g}=0.5$. The computation time and cost of each algorithm are shown in Table 5.

**Figure 12:**
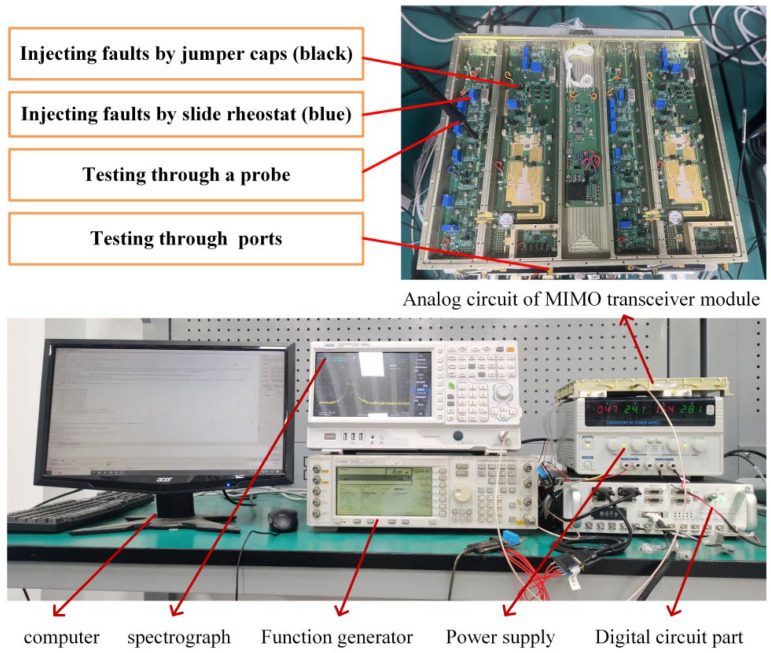
Two channels are used for testability design verification.

A two-channel MIMO circuit is selected as the second real case, as shown in Figure 12. This is the signal transmission and reception frontend module of a complex radar. Under the premise of ensuring a 100% fault isolation rate, a diagnosis strategy is required to isolate faults as quickly as possible. This system consists of 45 modules and 11 test points. Based on theoretical analysis and knowledge, all 73 faults of the system were identified by analyzing the possible fault modes of each module and their impact on system function. And 50 tests have been determined that can be used to detect all fault signals, as shown in the Appendix A.

**Figure 13:**
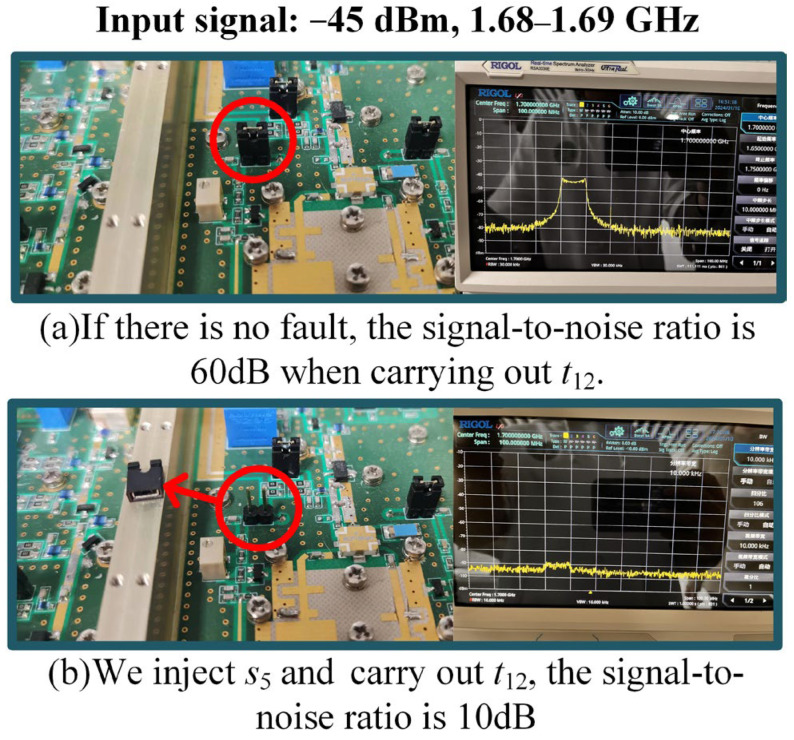
An example of fault injection.

Tests are performed by injecting signals at one test point (or two test points) and detecting the output at another test point. There are three methods for injecting faults, including plugging and unplugging jumper caps, rotating and sliding rheostats, and circuit simulation. The outputs before and after fault injection are compared to obtain D,P,C. An example is shown in Figure 13. We inject fault $s_{5}$ and carry out $t_{12}$; a decrease in the signal-to-noise ratio from 60 dB to 10 dB is detected while carrying out the symmetry test $t_{37}$. The signal-to-noise ratio is kept at 60 dB, so $d_{5,12}=1,d_{5,37}=0$. Information on the faults and tests, the system schematic diagram, and D are provided in the Appendix A.

Based on Experiments 1 and 2, PSO_MLP1 is selected with θ=4 and $r_{g}=0.5$. The computation time and cost of each algorithm are shown in Table 6.

Consistent with the simulation results, the cost of PSO_MLP1 outperforms that of the other algorithms in two real cases, and it yields more significant advantages in the second case D, which has a larger scale (only 63.9% of the Growing algorithm). The computation time of PSO_MLP1 is shorter than that of the QGA, HPSO, and Hybrid. And this advantage is not significant in the large system of the second case. Although the Growing algorithm has a shorter calculation time, the testability design does not have real-time requirements, so 26.045 s is completely acceptable. This demonstrates the effectiveness of the multipartite graph model and ENI structure. The strategy of PSO_MLP1 is shown in the Appendix A. Following this strategy for fault isolation, 300 faults are injected based on P. All the diagnostic results were consistent with those of the injection, and the average cost was consistent with the expected cost. This indicates that the proposed method can generate a better diagnosis strategy, which is feasible for improving testing efficiency and reducing comprehensive test costs.

In the current real-case verification, the injected fault samples were used to check the consistency between the observed test outcomes and the binary dependency matrix D. Since the proposed strategy is derived from D rather than learned from data, these samples serve as consistency-verification samples instead of training/testing samples in the conventional machine-learning sense.

The present study is based on a deterministic binary dependency matrix D. Such a formulation is appropriate for systems in which the test responses are highly repeatable and strongly separable as shown in (3), i.e., repeated executions of the same test under the same fault state produce stable binary outcomes. In cases where the discrimination capability of a test is weak or the test design is not sufficiently informative, the same test may provide only partial separation for certain faults, and the corresponding dependency relation is more appropriately represented by a fractional or probabilistic value between 0 and 1. Moreover, in more complex industrial scenarios, the outcome of a test may also be affected by sensor noise, measurement error, environmental disturbance, parameter drift, intermittent faults, or overlap between fault signatures, so that the same test under the same fault state does not always yield an identical result. This issue is especially relevant when the discrimination is based on waveform similarity, spectral features, or analog quantities near a threshold, where repeated tests may produce results that are distinguishable in most cases but not in all cases. Therefore, the difference between binary and probabilistic dependency relations reflects not only different forms of test-result representation, but also differences in test design quality and engineering application scenarios.

## 5. Conclusions

Based on the multi-signal flow graph model, minimizing the diagnostic cost is described as an MDP. Due to the combinatorial explosion, most existing algorithms are hard to solve this problem in complex systems. Therefore, this paper transforms the complex MDP into a simpler FEVP for nondeterministic elimination. Based on the ENI structure, the unknown function of FEVP is decomposed into three parts and solved separately. The simulation results show that different neural network structures and intelligent algorithms can establish the proposed ENI structure. Compared to existing algorithms, it offers greater advantages when applied to large-scale systems. The experiment results show that within an acceptable time, the cost can be reduced to 69.4% of comparison algorithms and can reach 63.9% on the testability design case of a two-channel MIMO system.

The present model is mainly suitable for testability-design problems with highly deterministic test responses. For systems with significant test uncertainty, the modeling stage itself should be adjusted by introducing probabilistic dependency matrices, robust strategy-generation rules, or joint cost–risk optimization criteria. In other words, whether test uncertainty should be explicitly incorporated into the MDP depends on the test mechanism, the signal-to-noise ratio, and the repeatability of the test outcomes in the target system. In this paper, we focus on the deterministic setting in order to clearly demonstrate the advantage of the multipartite graph model and the ENI structure for large-scale minimum-cost strategy generation, while the uncertainty-aware extension will be investigated in future work.

In our future work, we plan to implement the following three extensions to the proposed method: The first item is to improve the universality of the ENI structure and expand its application scope to the top-down and horizontal methods. The second item is to develop an adaptive method based on the ENI structure to improve the robustness of elimination action applied on different distributions of D. The third item is to integrate with test uncertainty modeling, so that the proposed framework can be extended to probabilistic or weighted test-response representations for more engineering scenarios.

## Figures and Tables

**Table 1:** The estimation value of $\left|{best}_{i}\right|$.

i	1	2	3	4	5	6	7	8
$\left|{best}_{i}\right|$	50	18.04	9.02	5.25	3.38	2.35	1.70	1.25
i	8	9	10	11	12	13	>14	
$\left|{best}_{i}\right|$	1.25	0.95	0.77	0.67	0.60	0.54	<0.50	

**Table 2:** ${∆}_{1}/{∆}_{2}\;$ under different m,n.

m,n	30	40	50	60	70	80
${∆}_{1}/{∆}_{2}$	0.0772	0.0816	0.0874	0.0917	0.1492	0.2435

**Table 3:** Normalized test cost and computation time (s) of existing algorithms.

Existing Algorithm				
m,n	QGA	HPSO	Growing	Hybrid
30	1.26/13.70	1.51/4.92	1.29/0.01	1.08/134.36
40	1.47/19.18	1.64/9.64	1.22/0.02	1.07/552.51
50	1.57/25.05	1.64/13.34	1.30/0.04	1.11/2761.98
60	1.56/31.71	1.93/18.48	1.31/0.06	1.05/>1 d
70	1.83/38.35	1.96/24.74	1.42/0.09	/>7 d
80	1.85/46.48	2.02/27.30	1.44/0.17	/>30 d

**Table 4:** Normalized test cost and computation time (s) of the proposed method.

Proposed Method (Various Intel-Ligent Algorithms and Neural Networks on the ENI)										
m,n.	θ	PSO MLP1	PSO MLP2	PSO LSTM	GA MLP1	GA MLP2	GA LSTM	CS MLP1	CS MLP2	CS LSTM
		$r_{g}=0.5$								
30	3	1.00/0.58	0.96/0.54	1.00/0.58	1.03/0.60	1.02/0.61	1.01/0.65	0.97/0.54	0.90/0.60	1.06/0.60
40	3	1.00/1.78	1.02/2.09	1.03/2.08	1.01/2.15	1.07/2.21	0.97/2.35	1.04/1.95	0.93/2.16	1.09/2.07
50	4	1.00/6.45	1.04/7.51	1.05/8.60	0.98/7.50	0.91/8.57	1.03/8.36	1.08/7.36	1.01/7.61	0.91/10.40
60	4	1.00/17.76	0.98/15.93	1.12/18.41	1.01/16.64	1.03/16.45	1.08/19.22	0.91/17.45	1.09/16.86	0.98/18.26
70	5	1.00/48.59	1.01/51.76	0.96/51.55	0.96/49.94	0.91/52.88	1.10/52.01	1.09/51.07	1.03/53.82	1.09/57.50
80	5	1.00/84.93	1.03/92.24	0.92/91.99	0.98/90.06	0.97/89.01	0.98/91.05	1.05/87.72	0.96/89.99	1.01/97.01

**Table 5:** The comparison results of the control moment gyro system.

Algorithm	Direction of Generating	FIR	Cost	Computation Time (s)	Peak CPU Usage
PSO_MLP1	bottom-up	100%	5.459	0.658	18.3%
QGA	top-down	100%	5.909	25.902	21.9%
HPSO	top-down	100%	5.939	5.699	22.8%
Growing	horizontal	100%	5.571	0.035	18.6%
Hybrid	bottom-up	100%	5.478	126.355	24.1%

**Table 6:** The comparison results of the MIMO system.

Algorithm	Direction of Generating	FIR	Cost	Computation Time (s)	Peak CPU Usage
PSO_MLP1	bottom-up	100%	3.480	26.045	17.8%
QGA	top-down	100%	5.521	34.889	22.5%
HPSO	top-down	100%	5.724	28.249	24.8%
Growing	horizontal	100%	5.444	0.092	20.7%
Hybrid	bottom-up	100%	3.537	>7 d	\

**Figure A1:**
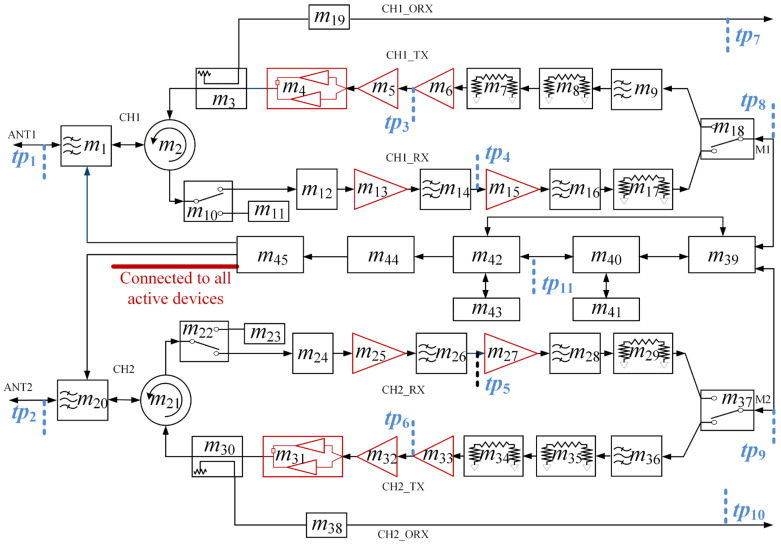
The system schematic diagram of two channels MIMO.

**Figure A2:**
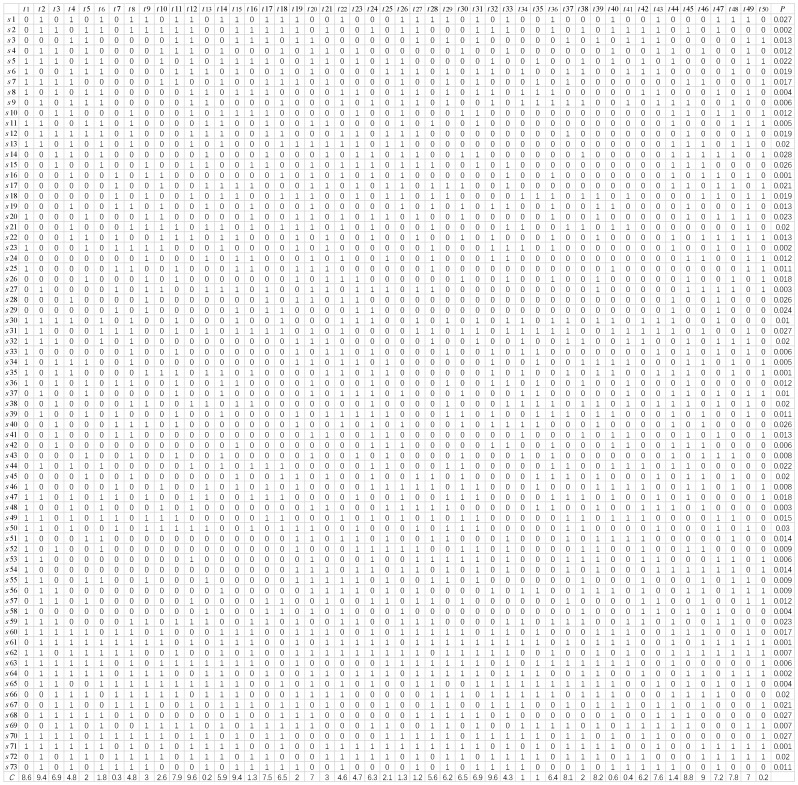
The D,P and C of the MIMO system.

## Data Availability

All the relevant data have been declared and provided in the manuscript.

## Appendix A

Based on the system schematic diagram of the two-channel MIMO circuit, all fault modules have been divided by analyzing the minimum replaceable units, and corresponding faults have been identified through theoretical analysis and expert knowledge. Module and fault information of the MIMO system is shown in Table A1.

**Table A1:** Module and fault information of the MIMO system.

Module	Module Name	Fault	Module	Module Name	Fault
$m_{1}$	cavity filter	$s_{1}$	$m_{24}$	limiter	$s_{34}$
$m_{2}$	circulator	$s_{2}$	$m_{25}$	low noise amplifier	$s_{35},s_{36}$
$m_{3}$	power divider	$s_{3},s_{4}$	$m_{26}$	RF filter	$s_{37}$
$m_{4}$	Doherty	$s_{5},s_{6},s_{7}$	$m_{27}$	low noise amplifier	$s_{38},s_{39}$
$m_{5}$	RF amplifier	$s_{8}$	$m_{28}$	RF bandpass filter	$s_{40},s_{41}$
$m_{6}$	driver amplifier	$s_{9}$	$m_{29}$	digital attenuator	$s_{42},s_{43}$
$m_{7}$	Temperature attenuator	$s_{10}$	$m_{30}$	power divider	$s_{44},s_{45}$
$m_{8}$	π-type attenuator	$s_{11},s_{12}$	$m_{31}$	Doherty	$s_{46},s_{47},s_{48}$
$m_{9}$	RF bandpass filter	$s_{13},s_{14}$	$m_{32}$	RF amplifier	$s_{49}$
$m_{10}$	RF switch	$s_{15}$	$m_{33}$	driver amplifier	$s_{50}$
$m_{11}$	RF load	$s_{16}$	$m_{34}$	Temperature attenuator	$s_{51}$
$m_{12}$	limiter	$s_{17}$	$m_{35}$	π-type attenuator	$s_{52},s_{53}$
$m_{13}$	low noise amplifier	$s_{18},s_{19}$	$m_{36}$	RF bandpass filter	$s_{54},s_{55}$
$m_{14}$	RF filter	$s_{20}$	$m_{37}$	RF switch	$s_{56}$
$m_{15}$	low noise amplifier	$s_{21},s_{22}$	$m_{38}$	digital attenuator	$s_{57},s_{58}$
$m_{16}$	RF bandpass filter	$s_{23},s_{24}$	$m_{39}$	broadband transceiver	$s_{59},s_{60}$
$m_{17}$	digital attenuator	$s_{25},s_{26}$	$m_{40}$	FPGA	$s_{61}s_{62},s_{63}$
$m_{18}$	RF switch	$s_{27}$	$m_{41}$	Memory	$s_{64}$
$m_{19}$	digital attenuator	$s_{28},s_{29}$	$m_{42}$	controller	$s_{65},s_{66},s_{67}$
$m_{20}$	cavity filter	$s_{30}$	$m_{43}$	Memory	$s_{68}$
$m_{21}$	circulator	$s_{31}$	$m_{44}$	Timing control module	$s_{69},s_{70}$
$m_{22}$	RF switch	$s_{32}$	$m_{45}$	Power module	$s_{71},s_{72},s_{73}$
$m_{23}$	RF load	$s_{33}$			

The test methods employed by the two-channel MIMO circuit system are all active tests. By injecting excitation signals at TPA and detecting outputs at TPB, circuit signal detection is achieved. Test information of the MIMO system is shown in Table A2.

**Table A2:** Test information of the MIMO system.

	TPA	TPB	Test Name		TPA	TPB	Test Name
$t_{1}$	${tp}_{8},{tp}_{9}$	${tp}_{6}$	main-to-noise ratio of output signal	$t_{26}$	${tp}_{9},{tp}_{8}$	${tp}_{3}$	main-to-noise ratio of output signal
$t_{2}$	${tp}_{8},{tp}_{9}$	${tp}_{2}$	noise factor	$t_{27}$	${tp}_{9},{tp}_{8}$	${tp}_{1}$	noise factor
$t_{3}$	${tp}_{8}$	${tp}_{5}$	isolation degree	$t_{28}$	${tp}_{9}$	${tp}_{4}$	isolation degree
$t_{4}$	${tp}_{8}$	${tp}_{2}$	isolation degree	$t_{29}$	${tp}_{9}$	${tp}_{1}$	isolation degree
$t_{5}$	${tp}_{8}$	${tp}_{5}$	coupling degree	$t_{30}$	${tp}_{9}$	${tp}_{4}$	coupling degree
$t_{6}$	${tp}_{8}$	${tp}_{2}$	coupling degree	$t_{31}$	${tp}_{9}$	${tp}_{1}$	coupling degree
$t_{7}$	${tp}_{1},{tp}_{2}$	${tp}_{6}$	main-to-noise ratio of output signal	$t_{32}$	${tp}_{1},{tp}_{2}$	${tp}_{3}$	main-to-noise ratio of output signal
$t_{8}$	${tp}_{1},{tp}_{2}$	${tp}_{4}$	noise factor	$t_{33}$	${tp}_{1},{tp}_{2}$	${tp}_{5}$	noise factor
$t_{9}$	${tp}_{1}$	${tp}_{9}$	gain	$t_{34}$	${tp}_{2}$	${tp}_{8}$	gain
$t_{10}$	${tp}_{1}$	${tp}_{6}$	SWR	$t_{35}$	${tp}_{2}$	${tp}_{3}$	SWR
$t_{11}$	${tp}_{1}$	${tp}_{5}$	dynamic range	$t_{36}$	${tp}_{2}$	${tp}_{4}$	dynamic range
$t_{12}$	${tp}_{8}$	${tp}_{1}$	signal-to-noise ratio	$t_{37}$	${tp}_{9}$	${tp}_{2}$	signal-to-noise ratio
$t_{13}$	${tp}_{8}$	${tp}_{3}$	gain	$t_{38}$	${tp}_{9}$	${tp}_{6}$	gain
$t_{14}$	${tp}_{8}$	${tp}_{4}$	Spurious suppression	$t_{39}$	${tp}_{9}$	${tp}_{5}$	spurious suppression
$t_{15}$	${tp}_{1}$	${tp}_{3}$	insertion loss	$t_{40}$	${tp}_{2}$	${tp}_{6}$	insertion loss
$t_{16}$	${tp}_{1}$	${tp}_{4}$	frequency range	$t_{41}$	${tp}_{2}$	${tp}_{5}$	frequency range
$t_{17}$	${tp}_{8}$	${tp}_{7}$	power	$t_{42}$	${tp}_{9}$	${tp}_{10}$	power
$t_{18}$	${tp}_{8}$	${tp}_{10}$	signal-to-noise ratio	$t_{43}$	${tp}_{9}$	${tp}_{7}$	signal-to-noise ratio
$t_{19}$	${tp}_{8},{tp}_{9}$	${tp}_{11}$	data stream detection 1	$t_{44}$	${tp}_{1},{tp}_{2}$	${tp}_{11}$	data stream detection 7
$t_{20}$	${tp}_{8}$	${tp}_{11}$	data stream detection 2	$t_{45}$	${tp}_{9}$	${tp}_{11}$	data stream detection 8
$t_{21}$	${tp}_{1}$	${tp}_{11}$	data stream detection 3	$t_{46}$	${tp}_{2}$	${tp}_{11}$	data stream detection 9
$t_{22}$	${tp}_{11}$	${tp}_{8},{tp}_{9}$	data stream detection 4	$t_{47}$	${tp}_{11}$	${tp}_{1},{tp}_{2}$	data stream detection 10
$t_{23}$	${tp}_{11}$	${tp}_{8}$	data stream detection 5	$t_{48}$	${tp}_{11}$	${tp}_{9}$	data stream detection 11
$t_{24}$	${tp}_{11}$	${tp}_{1}$	data stream detection 6	$t_{49}$	${tp}_{11}$	${tp}_{2}$	data stream detection 12
$t_{25}$	${tp}_{1}$	${tp}_{10}$	SWR	$t_{50}$	${tp}_{2}$	${tp}_{7}$	SWR

The system schematic diagram of two-channel MIMO is shown in Figure A1.

The diagnosis strategy is shown in Table A3, where the i-th row represents the tests that need to be performed sequentially to isolate *s_i_*. Taking isolation *s*_1_ as an example, *t*_13_, *t*_7_, *t*_41_, *t*_40_, *t*_50_, *t*_26_ are required sequentially.

**Table A3:** The diagnosis strategy of PSO_MLP1.

$s_{1}$	$t_{13}$	$t_{7}$	$t_{41}$	$t_{40}$	$t_{50}$	$t_{26}$			
$s_{2}$	$t_{13}$	$t_{7}$	$t_{41}$	$t_{50}$	$t_{40}$	$t_{16}$	$t_{27}$		
$s_{3}$	$t_{13}$	$t_{7}$	$t_{41}$	$t_{50}$	$t_{34}$	$t_{40}$			
$s_{4}$	$t_{13}$	$t_{7}$	$t_{41}$	$t_{40}$	$t_{50}$	$t_{26}$	$t_{27}$		
$s_{5}$	$t_{13}$	$t_{7}$	$t_{41}$	$t_{40}$	$t_{50}$	$t_{34}$			
$s_{6}$	$t_{13}$	$t_{7}$	$t_{41}$	$t_{40}$	$t_{35}$	$t_{16}$			
$s_{7}$	$t_{13}$	$t_{7}$	$t_{41}$	$t_{40}$	$t_{50}$	$t_{26}$	$t_{35}$		
$s_{8}$	$t_{13}$	$t_{7}$	$t_{41}$	$t_{50}$	$t_{16}$	$t_{35}$			
$s_{9}$	$t_{13}$	$t_{7}$	$t_{41}$	$t_{40}$	$t_{35}$	$t_{27}$			
$s_{10}$	$t_{13}$	$t_{7}$	$t_{41}$	$t_{40}$	$t_{50}$	$t_{26}$	$t_{35}$	$t_{27}$	
$s_{11}$	$t_{13}$	$t_{7}$	$t_{41}$	$t_{50}$	$t_{27}$	$t_{44}$	$t_{40}$		
$s_{12}$	$t_{13}$	$t_{7}$	$t_{41}$	$t_{50}$	$t_{16}$	$t_{27}$			
$s_{13}$	$t_{13}$	$t_{7}$	$t_{41}$	$t_{40}$	$t_{50}$	$t_{27}$	$t_{26}$		
$s_{14}$	$t_{13}$	$t_{7}$	$t_{41}$	$t_{50}$	$t_{27}$	$t_{35}$			
$s_{15}$	$t_{13}$	$t_{7}$	$t_{41}$	$t_{50}$	$t_{16}$	$t_{35}$			
$s_{16}$	$t_{13}$	$t_{7}$	$t_{50}$	$t_{41}$	$t_{35}$	$t_{34}$	$t_{26}$		
$s_{17}$	$t_{13}$	$t_{7}$	$t_{50}$	$t_{40}$	$t_{41}$	$t_{27}$			
$s_{18}$	$t_{13}$	$t_{7}$	$t_{50}$	$t_{41}$	$t_{40}$	$t_{35}$			
$s_{19}$	$t_{13}$	$t_{7}$	$t_{50}$	$t_{41}$	$t_{40}$	$t_{35}$			
$s_{20}$	$t_{13}$	$t_{7}$	$t_{41}$	$t_{40}$	$t_{50}$	$t_{27}$	$t_{16}$		
$s_{21}$	$t_{13}$	$t_{7}$	$t_{41}$	$t_{40}$	$t_{50}$				
$s_{22}$	$t_{13}$	$t_{7}$	$t_{50}$	$t_{41}$	$t_{40}$				
$s_{23}$	$t_{13}$	$t_{7}$	$t_{50}$	$t_{41}$	$t_{35}$	$t_{34}$			
$s_{24}$	$t_{13}$	$t_{7}$	$t_{41}$	$t_{50}$	$t_{27}$	$t_{44}$			
$s_{25}$	$t_{13}$	$t_{7}$	$t_{50}$	$t_{41}$	$t_{40}$				
$s_{26}$	$t_{13}$	$t_{7}$	$t_{41}$	$t_{40}$	$t_{50}$	$t_{26}$	$t_{27}$		
$s_{27}$	$t_{13}$	$t_{7}$	$t_{50}$	$t_{40}$	$t_{41}$	$t_{27}$			
$s_{28}$	$t_{13}$	$t_{7}$	$t_{41}$	$t_{40}$	$t_{50}$	$t_{27}$	$t_{26}$	$t_{34}$	
$s_{29}$	$t_{13}$	$t_{7}$	$t_{50}$	$t_{41}$	$t_{35}$	$t_{34}$	$t_{26}$		
$s_{30}$	$t_{13}$	$t_{7}$	$t_{41}$	$t_{40}$	$t_{50}$	$t_{26}$	$t_{27}$	$t_{34}$	
$s_{31}$	$t_{13}$	$t_{7}$	$t_{50}$	$t_{41}$	$t_{35}$				
$s_{32}$	$t_{13}$	$t_{7}$	$t_{41}$	$t_{50}$	$t_{40}$				
$s_{33}$	$t_{13}$	$t_{7}$	$t_{41}$	$t_{40}$	$t_{50}$	$t_{27}$	$t_{26}$	$t_{34}$	$t_{35}$
$s_{34}$	$t_{13}$	$t_{7}$	$t_{41}$	$t_{50}$	$t_{34}$	$t_{40}$			
$s_{35}$	$t_{13}$	$t_{7}$	$t_{41}$	$t_{50}$	$t_{27}$	$t_{44}$	$t_{40}$		
$s_{36}$	$t_{13}$	$t_{7}$	$t_{50}$	$t_{41}$	$t_{40}$				
$s_{37}$	$t_{13}$	$t_{7}$	$t_{41}$	$t_{50}$	$t_{34}$	$t_{26}$			
$s_{38}$	$t_{13}$	$t_{7}$	$t_{41}$	$t_{40}$	$t_{35}$	$t_{27}$			
$s_{39}$	$t_{13}$	$t_{7}$	$t_{50}$	$t_{41}$	$t_{35}$	$t_{40}$			
$s_{40}$	$t_{13}$	$t_{7}$	$t_{50}$	$t_{41}$	$t_{35}$	$t_{40}$			
$s_{41}$	$t_{13}$	$t_{7}$	$t_{50}$	$t_{41}$	$t_{40}$	$t_{16}$			
$s_{42}$	$t_{13}$	$t_{7}$	$t_{41}$	$t_{50}$	$t_{40}$	$t_{16}$	$t_{26}$		
$s_{43}$	$t_{13}$	$t_{7}$	$t_{41}$	$t_{40}$	$t_{50}$	$t_{27}$	$t_{26}$	$t_{34}$	$t_{35}$
$s_{44}$	$t_{13}$	$t_{7}$	$t_{41}$	$t_{50}$	$t_{40}$	$t_{16}$	$t_{27}$		
$s_{45}$	$t_{13}$	$t_{7}$	$t_{41}$	$t_{40}$	$t_{50}$	$t_{27}$	$t_{16}$	$t_{26}$	
$s_{46}$	$t_{13}$	$t_{7}$	$t_{50}$	$t_{40}$	$t_{34}$	$t_{41}$			
$s_{47}$	$t_{13}$	$t_{7}$	$t_{50}$	$t_{40}$	$t_{34}$	$t_{41}$			
$s_{48}$	$t_{13}$	$t_{7}$	$t_{41}$	$t_{40}$	$t_{50}$	$t_{27}$	$t_{16}$	$t_{26}$	$t_{35}$
$s_{49}$	$t_{13}$	$t_{7}$	$t_{50}$	$t_{41}$	$t_{40}$	$t_{35}$			
$s_{50}$	$t_{13}$	$t_{7}$	$t_{50}$	$t_{41}$	$t_{40}$				
$s_{51}$	$t_{13}$	$t_{7}$	$t_{41}$	$t_{50}$	$t_{40}$	$t_{16}$	$t_{26}$		
$s_{52}$	$t_{13}$	$t_{7}$	$t_{41}$	$t_{40}$	$t_{50}$	$t_{27}$	$t_{16}$	$t_{26}$	$t_{35}$
$s_{53}$	$t_{13}$	$t_{7}$	$t_{41}$	$t_{40}$	$t_{50}$	$t_{26}$	$t_{27}$	$t_{38}$	
$s_{54}$	$t_{13}$	$t_{7}$	$t_{41}$	$t_{40}$	$t_{50}$	$t_{26}$	$t_{35}$	$t_{27}$	
$s_{55}$	$t_{13}$	$t_{7}$	$t_{41}$	$t_{50}$	$t_{16}$	$t_{27}$	$t_{26}$		
$s_{56}$	$t_{13}$	$t_{7}$	$t_{41}$	$t_{40}$	$t_{50}$	$t_{34}$			
$s_{57}$	$t_{13}$	$t_{7}$	$t_{41}$	$t_{40}$	$t_{50}$	$t_{26}$	$t_{27}$	$t_{38}$	
$s_{58}$	$t_{13}$	$t_{7}$	$t_{41}$	$t_{50}$	$t_{16}$	$t_{27}$	$t_{26}$		
$s_{59}$	$t_{13}$	$t_{7}$	$t_{41}$	$t_{40}$	$t_{35}$	$t_{16}$			
$s_{60}$	$t_{13}$	$t_{7}$	$t_{50}$	$t_{41}$	$t_{40}$	$t_{35}$			
$s_{61}$	$t_{13}$	$t_{7}$	$t_{50}$	$t_{41}$	$t_{40}$	$t_{16}$	$t_{35}$		
$s_{62}$	$t_{13}$	$t_{7}$	$t_{50}$	$t_{41}$	$t_{40}$	$t_{35}$			
$s_{63}$	$t_{13}$	$t_{7}$	$t_{41}$	$t_{40}$	$t_{50}$				
$s_{64}$	$t_{13}$	$t_{7}$	$t_{41}$	$t_{50}$	$t_{27}$	$t_{35}$	$t_{40}$		
$s_{65}$	$t_{13}$	$t_{7}$	$t_{50}$	$t_{41}$	$t_{40}$	$t_{35}$	$t_{27}$		
$s_{66}$	$t_{13}$	$t_{7}$	$t_{50}$	$t_{41}$	$t_{40}$	$t_{35}$	$t_{27}$		
$s_{67}$	$t_{13}$	$t_{7}$	$t_{50}$	$t_{41}$	$t_{40}$	$t_{16}$	$t_{35}$		
$s_{68}$	$t_{13}$	$t_{7}$	$t_{41}$	$t_{40}$	$t_{50}$	$t_{26}$	$t_{27}$	$t_{34}$	
$s_{69}$	$t_{13}$	$t_{7}$	$t_{41}$	$t_{50}$	$t_{34}$	$t_{26}$			
$s_{70}$	$t_{13}$	$t_{7}$	$t_{50}$	$t_{40}$	$t_{34}$				
$s_{71}$	$t_{13}$	$t_{7}$	$t_{41}$	$t_{50}$	$t_{27}$	$t_{35}$	$t_{40}$		
$s_{72}$	$t_{13}$	$t_{7}$	$t_{50}$	$t_{40}$	$t_{41}$				
$s_{73}$	$t_{13}$	$t_{7}$	$t_{50}$	$t_{41}$	$t_{35}$				

The D,P and C of the MIMO system is shown in Figure A2.
